# CSearch: chemical space search via virtual synthesis and global optimization

**DOI:** 10.1186/s13321-024-00936-8

**Published:** 2024-12-05

**Authors:** Hakjean Kim, Seongok Ryu, Nuri Jung, Jinsol Yang, Chaok Seok

**Affiliations:** 1 Department of Chemistry, Seoul National University, Seoul, 08826 Republic of Korea; 2 Galux Inc, Seoul, 08738 Republic of Korea

**Keywords:** Chemical space search, Computer-aided drug design, Global optimization, Virtual synthesis

## Abstract

The two key components of computational molecular design are virtually generating molecules and predicting the properties of these generated molecules. This study focuses on an effective method for molecular generation through virtual synthesis and global optimization of a given objective function. Using a pre-trained graph neural network (GNN) objective function to approximate the docking energies of compounds for four target receptors, we generated highly optimized compounds with 300–400 times less computational effort compared to virtual compound library screening. These optimized compounds exhibit similar synthesizability and diversity to known binders with high potency and are notably novel compared to library chemicals or known ligands. This method, called CSearch, can be effectively utilized to generate chemicals optimized for a given objective function. With the GNN function approximating docking energies, CSearch generated molecules with predicted binding poses to the target receptors similar to known inhibitors, demonstrating its effectiveness in producing drug-like binders.

**Scientific Contribution** We have developed a method for effectively exploring the chemical space of drug-like molecules using a global optimization algorithm with fragment-based virtual synthesis. The compounds generated using this method optimize the given objective function efficiently and are synthesizable like commercial library compounds. Furthermore, they are diverse, novel drug-like molecules with properties similar to known inhibitors for target receptors.

**Supplementary Information:**

The online version contains supplementary material available at 10.1186/s13321-024-00936-8.

## Introduction

In silico molecular discovery and optimization techniques are highly anticipated due to recent advancements in artificial intelligence (AI) technology. Typically, in silico molecular design of drug-like properties against specific target proteins involves two components: generating candidate molecules with desired properties and predicting the properties of the given molecules. Deep learning techniques related to molecular design are being developed independently for drug-like molecular generation [1–3] and precise molecular property prediction [4–7]. This is because developing each technology is both challenging and applicable to diverse areas. In the future, combining these two technologies will become an important issue.

One recent example of successful integration of molecular generation and property prediction in molecular design is the synthon-based approach [8], which uses a virtual reaction-based, non-AI molecular generation method and a non-AI docking method for property prediction. This method gradually optimizes molecules by sequentially increases their size through virtual reactions and screening the molecular library generated at each step with docking.

The molecular generation method using virtual reactions [9, 10] effectively ensures the chemical validity and synthesizability of the molecules, compared to methods that generate molecules in a latent space of virtual compounds [11, 12]. Generative methods like diffusion models [13–15] and reinforcement learning [3, 11, 16, 17] have recently been proposed to generate chemically valid compounds with desired properties. In these methods, objective functions such as QED (Quantitative Estimate of Drug-likeness) [18], log P (a measure of a compound’s hydrophobicity), SA (Synthetic Accessibility) [19], a linear combination of them, or reward model for molecular properties were integrated.

Accurate evaluation of both technical components—molecular generation and property prediction—used in molecular design methods would help in assessing and advancing the design methods. Molecular property prediction models such as binding affinity or toxicity prediction models [4–6, 20–22] can be evaluated by comparing their predictions with experimental data [7, 23], but molecular generation models are often evaluated in conjunction with different property prediction models [24–28], making the objective comparison difficult. If molecular generation models could be objectively evaluated and improved, it would be significant for the advancement of molecular design technology.

In this study, we address the molecular generation problem as a task of optimizing a given objective function and provide a case for comparative evaluation of molecular optimization performance. We used surrogate GNN models to approximate the docking scores for four protein receptors as a realistic yet computationally inexpensive example objective function. A new molecular optimization, CSearch, is introduced by extending the global optimization method, conformational space annealing (CSA) [29], previously used for molecular structure prediction [30–34], to compound space optimization. Unlike the previously reported molecule generation method applying CSA [35], which focuses on optimizing only QED and SA in the chemical space represented by SMILES, CSearch optimizes drug-target-specific objective functions in the chemical space of synthesizable compounds generated through virtual reactions [36].

CSearch was compared with the virtual screening method of a 10^6^ library of drug-like molecules and a reinforcement learning-based chemical generation method, REINVENT4 [37] for four receptors. We confirmed that the molecular optimization performance of CSearch was at least 300–400 times more computationally efficient. The synthesizability and diversity of the highly optimized compounds generated by CSearch were only slightly lower than those of the less optimized compounds obtained through library screening and were similar to known ligands with high potency. Additionally, the optimized compounds were significantly more novel compared to library chemicals or known ligands.

These results demonstrate that the new chemical optimization method, CSearch, can serve as a robust baseline model for generating molecules optimized for a given objective function, thereby facilitating the development of more advanced molecular generation and optimization models. As more accurate molecular property prediction models become available, they can be incorporated into CSearch as objectives. Additionally, CSearch is a powerful molecular generation model with the potential to be extended to consider multiple properties simultaneously in a multi-objective manner. CSearch is freely available at the provided link: https://github.com/seoklab/CSearch.

## Methods

### Overview of CSearch: global optimization in the chemical space

**Fig. 1:**
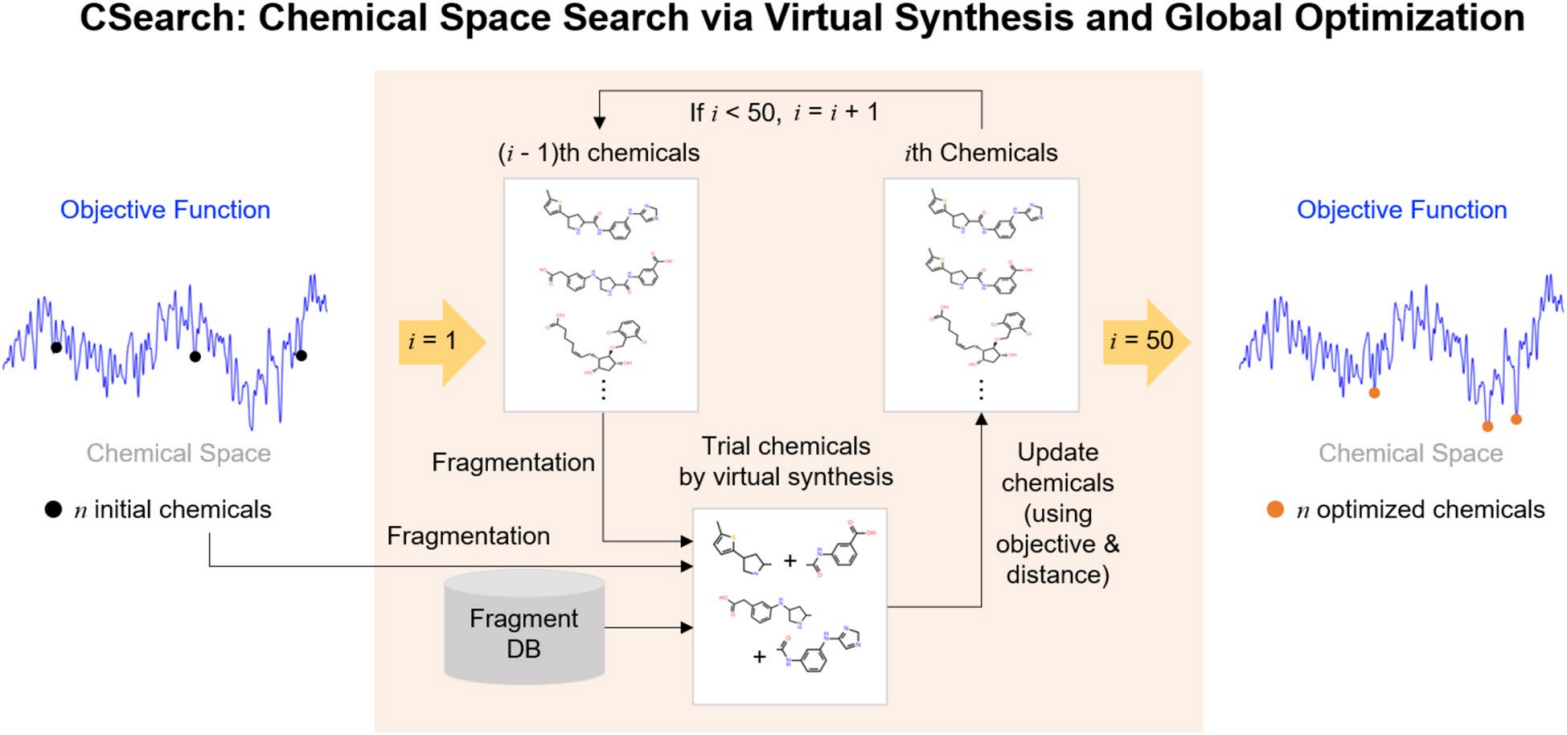
Overview of the CSearch workflow. Global optimization of the given objective function is performed in the chemical space by evolving a chemical bank consisting of a fixed number of chemicals. This iterative process involves generating trial chemicals through virtual synthesis using chemical fragments and updating the bank based on their objective values and distances

As shown in Fig. 1, CSearch takes a fixed number, *n*, of diverse initial chemicals, called the initial bank, and generates an equal number of optimized chemicals, called the final bank, based on a given objective function. During the global optimization of the objective function, trial chemicals are iteratively generated by virtual synthesis from the (*i* -1)th bank (the (*i* -1)th set of chemicals), the initial bank, and an external fragment database. The bank is updated *i*th bank based on the objective values and distances of the trial chemicals compared to the (*i* -1)th bank chemicals, following the principles of conformational space annealing [29]. This process results in globally optimal and sub-optimal chemicals in terms of the objective function. To evaluate the effectiveness of this chemical search method, we employed four GNNs trained to reproduce docking energies for four protein targets, along with BRICS rules [36] for virtual chemical fragmentation and synthesis. Further details of the method are described in the subsequent subsections.

### Global chemical optimization by chemical space annealing

The global optimization algorithm, conformational space annealing, has previously been used for optimizing objective functions within conformational space for structure prediction [29–34]. We now extend this approach to Chemical Space Annealing (CSA) to optimize objective function in the chemical space.

Since the initial chemicals and the fragment database, shown in Fig. 1, contribute to chemical diversity during the search process, careful attention was given to utilizing this diversity during chemical generation. We curated a pool of 1217 non-redundant, drug-like molecules from 2216 DrugspaceX [38] molecules by clustering with a Tanimoto similarity threshold [39] (calculated from Morgan Fingerprint [40] by RDkit [41] of 0.7. The initial bank of *n* = 60 molecules with the best objective function values was selected from this curated pool. The fragment database consists of 192,498 non-redundant fragments curated from the Enamine Fragment Collection [42], with a maximum Tanimoto similarity of 0.7 between fragments.

Each chemical in the bank of *n* = 60 is regarded as a representative within a radius *R*_cut_ in the chemical space. The distance between compounds is measured using Tanimoto similarity subtracted from 1. The initial *R*_cut_ is set to half of the average distance among the initial bank chemicals. The initial *R*_cut_ values were 0.423, 0.426, 0.428 and 0.425 for the four receptors introduced in Methods 2.3.

This radius is gradually reduced by a factor of 0.4^0.05^ at each CSA cycle, reaching 40% of the initial *R*_cut_ after 20 cycles and then kept constant in subsequent cycles. This strategy induces an effective global optimization by starting with a broad exploration of the chemical space and gradually transitioning to a more focused search in later cycles.

**Fig. 2:**
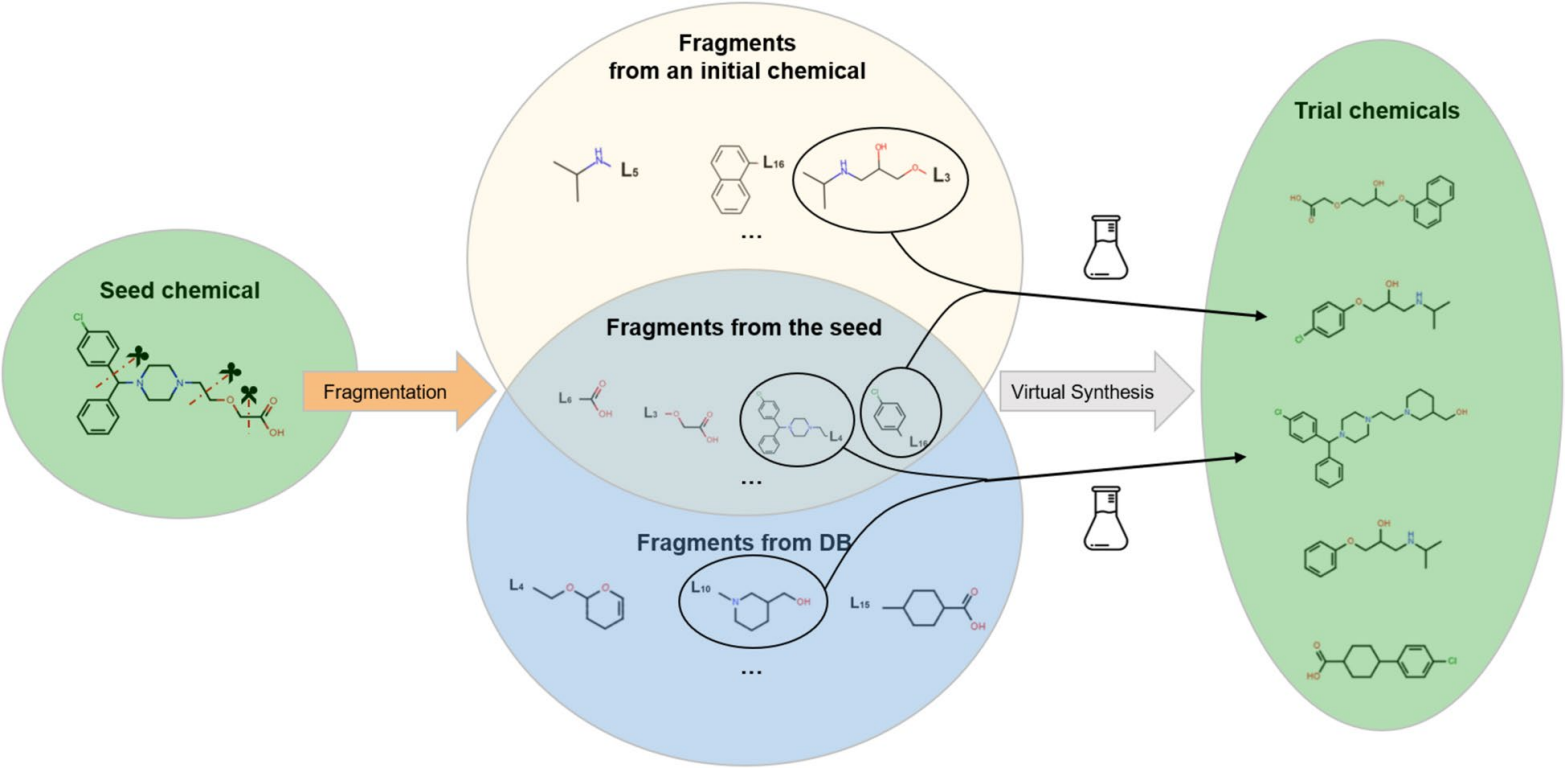
Trial chemical generation from a seed chemical. Fragments generated from the seed chemical are combined with fragments from an initial bank chemical and fragments from the database to generate trial chemicals. These fragmentation mechanisms are based on BRICS retrosynthesis rules, which consider 16 chemical environments

Each CSA cycle consists of generating trial chemicals from seed chemicals and updating the bank at a fixed *R*_cut_. For each of the six seed chemicals randomly selected from those not used as seeds in the current bank, trial chemicals are synthesized, as illustrated in Fig. 2. Virtual synthesis is performed by fragmenting each chemical and combining two fragments, as detailed in the next paragraph. Up to 60 chemicals are synthesized from a seed chemical and a randomly selected initial bank chemical, and up to 60 more chemicals are synthesized from the seed chemical and a randomly selected set of 100 fragments from the fragment database. A trial chemical replaces the nearest bank chemical within *R*_cut_ if it has a better objective value or replaces the bank chemical with the worst objective value if it is further away than *R*_cut_ from all bank members. Otherwise, the trial chemical is discarded. This procedure, from trial chemical generation to bank update, is repeated until all bank chemicals are used as seeds. The entire cycle is repeated once more after all bank chemicals are reset to unused. CSA is terminated after 50 such cycles. The specific CSA parameter values mentioned above were determined through prior parameter optimization. (Additional file 1: Method S1.)

Fragmentation of a chemical is performed by generating all possible fragments of more than three atoms with a single reaction point based on the BRICS rules, which defines 16 types of reaction points [36] (Additional file 1: Figure S1). Virtual synthesis is performed by matching a fragment from the seed chemical with a partner fragment that satisfies the BRICS synthesis rules. Each fragment is selected with a probability proportional to the average log frequency of each fragment’s Morgan Fingerprint in the PubChem database [40, 43]. This fragment selection strategy for virtual synthesis was chosen to improve the synthetic accessibility (SA) score by accounting for fragment distribution biases found in lab-synthesized chemicals (See Table S4).

### Objective functions employed to test CSearch

Four objective functions were developed to estimate the binding of a given chemical to each of four different receptors: SARS-CoV-2 main protease (MPro), tyrosine-protein kinase BTK (BTK), anaplastic lymphoma kinase (ALK), and H1N1 neuraminidase (H1N1_NA). These functions were created by regressing the GalaxyDock3 [44] docking energy with a GNN, enabling fast evaluation of the objective function.

The training, validation, and test sets for the regression task were obtained by randomly splitting a set of 10^6^ molecules from ChEMBL27 database [45] into a 7:1:2 ratios. For each receptor-chemical pair, the GalaxyDock3 [44] docking energy was obtained by performing docking calculations using the protein structure from the RCSB PDB [46] (PDB IDs 6m0k, 5p9h, 4mkc, and 3ti5 for MPro, BTK, ALK, and H1N1_NA, respectively).

The same GNN architecture was used for the four receptors (Additional file 1: Method S2). An estimated docking score is generated from the graph representation of the input molecule. Nodes are assigned to the heavy atoms, and edges are assigned to the chemical bonds (Additional file 1: Table S1). The resulting GNN models show high *R*^2^ values of 0.872, 0.836, 0.826, and 0.863 on the test set for MPro, BTK, ALK, and H1N1_NA, respectively (Additional file 1: Table S3).

### Comparison of CSearch with virtual library screening and REINVENT4, a reinforcement learning generation method

The performance of CSearch was evaluated in terms of its efficiency in optimizing the objective function and generating a diverse set of novel and synthesizable chemicals, compared to virtual screening and a reinforcement learning-based chemical generation method, REINVENT4 [37]. REINVENT4 was chosen for comparison because it allows for the use of a user-specified objective function built on the pre-trained model ‘Mol2Mol’.

A virtual chemical library screening was performed on a set of 1,352,699 drug-like molecules from the Enamine HTS Collection [47], with the top 300 compounds selected for comparison. The CSA optimization of CSearch was conducted 5 times using the same set of 60 initial compounds, generating 60 compounds per CSA run to reach a total of 300. Similarly, REINVENT4 was run 60 times with the same initial compounds as CSearch, and the top 300 compounds were selected for comparison. For all three methods, the same objective function was applied across each of the four receptors.

To establish a baseline for the background properties of non-optimized chemicals, we compared the properties of 2000 randomly selected chemicals from the 2206 chemicals in DrugspaceX [38], 2000 randomly selected chemicals from ZINC tranches [48–50] after drug-likeness filtering (250 < M.W. < 500, log P < 5, and Lipinski’s rule of 5 [51]), and 300 known binders with the best IC_50_ for each of the four receptors from BindingDB [52, 53].

Synthesizability was measured by the synthetic accessibility score [19], and this score is referred to as Synthetic Accessibility (SA) score, where a higher score indicates lower synthesizability. Chemical novelty was examined in t-SNE dimensions derived from 2000 randomly selected compounds from each of the Enamine HTS Collection, DrugspaceX, and ZINC databases, along with 300 known binders from BindingDB and 300 compounds generated by both CSearch and REINVENT4, using Morgan Fingerprints with parameters detailed in Additional file 1 (Method S2).

## Results and discussion

### Optimization efficiency of CSearch in comparison to virtual screening and REINVENT4

The efficiency of chemical optimization achieved by CSearch was compared with a virtual chemical screening performed on the Enamine HTS Collection and a reinforcement learning-based optimization method REINVENT4 [37], based on the objective values (predicted docking scores) of the optimized chemicals, as shown in Table 1. The number of objective function evaluations and runtime are presented in Table 2.

**Table 1:** Comparison of optimized objective values and average of the top 300 values for virtual screening, REINVENT4, and CSearch

Objective function	Virtual screening	REINVENT4	CSearch
MPro	Initial: − 113.8 (− 82.9)		
	− 149.3 (− 122.3)	**− 157.6** (− 133.5)	− 156.0 (**− 139.4**)
BTK	Initial: − 166.7 (− 96.4)		
	**− 202.4** (− 151.4)	− 187.2 (− 142.2)	− 199.6 (**− 184.7**)
ALK	Initial: − 113.4 (− 79.9)		
	− 133.8 (− 114.6)	− 149.2 (− 124.9)	**− 150.4** (**− 144.7**)
H1N1 NA	Initial: − 113.7 (− 79.5)		
	− 131.0 (− 113.2)	− 136.6 (− 119.6)	**− 148.7** (**− 140.6**)

The bold number in the row is the best value of the objective function

Initial values, top value and average of the top 60, are also presented

**Table 2:** Number of objective function evaluations and runtime in parentheses for virtual screening, REINVENT4, and CSearch

Objective function	Virtual screening^a^	REINVENT4^b^	CSearch^a^
MPro	1.35 × 10^6^ (2.93 × 10^3^ s)	1.34 × 10^5^ (2.31 × 10^3^ s)	**3.40 × 10**^**3**^ (**3.39 × 10**^**2**^** s**)
BTK	1.35 × 10^6^ (5.84 × 10^3^ s)	1.33 × 10^5^ (2.16 × 10^3^ s)	**2.64 × 10**^**3**^** (3.13 × 10**^**2**^** s**)
ALK	1.35 × 10^6^ (2.17 × 10^3^ s)	1.32 × 10^5^ (2.01 × 10^3^ s)	**3.52 × 10**^**3**^** (3.54 × 10**^**2**^** s**)
H1N1 NA	1.35 × 10^6^ (2.18 × 10^3^ s)	1.34 × 10^5^ (2.18 × 10^3^ s)	**3.74 × 10**^**3**^** (4.03 × 10**^**2**^** s**)

The bold number in the row is the best value of the objective function

^a^Executed on a single Intel Xeon Gold 6248R

^b^Executed on an NVIDIA RTX A6000

It can be seen from Tables 1 and 2 that CSearch can generate chemicals with more optimal (more negative) objective values, both the top 1 and the average of the top 300, with 300–400 times fewer objective evaluations compared to screening a library of size 1,352,699 for all four objective functions, except for BTK, where a better top 1 objective value was obtained with library screening. CSearch is also more efficient than REINVENT4 in both optimizing the objective function and reducing the number of objective evaluations, except for MPro, where REINVENT4 achieves a slightly better top 1 objective value. The runtime for CSearch is also significantly shorter than for both virtual screening and REINVENT4 (with CSearch and virtual screening run on CPU and REINVENT4 on GPU). The gain in runtime can be significant if the objective evaluation requires extensive computation, such as free energy calculation based on molecular dynamics simulations. This result illustrates the high potential of CSearch in solving the chemical optimization problem when an accurate objective function is available.

In the next subsection, the effectiveness of CSearch’s chemical generation component, specifically the BRICS-based method, is evaluated. The use of a surrogate objective function, applied as a predicted docking score, is also discussed. Subsequently, the synthesizability, diversity, and novelty of the chemicals generated by CSearch are reported.

### Effectiveness of the BRICS-based virtual synthesis and the surrogate objective function

To evaluate the effectiveness of the BRICS-rule-based virtual synthesis used in CSA global optimization, we assess how well a reference molecule can be recovered by using a structure-alignment score, termed G-Align, as an objective function for optimization. The G-Align score, defined below, measures the fraction of atomic overlap between the reference and query molecule on a scale from 0 to 1, with 1 indicating identity, after flexible structure alignment using CSAlign [54].

$$\text{G}-\text{Align}=\frac{{\sum }_{i\in Q}{\sum }_{j\in R}{V}_{QR}^{ij}}{\text{max}({\sum }_{i\in Q}{\sum }_{j\in Q}{V}_{QQ}^{ij},{\sum }_{i\in R}{\sum }_{j\in R}{V}_{RR}^{ij})}$$

where ${V}_{QR}^{ij}$, ${V}_{QQ}^{ij}$, ${V}_{RR}^{ij}$ refer to the volume of the intersection between the spheres of the* i*-th query (Q) or reference (R) atom and the *j*-th query (Q) or reference (R) atom. The radius of each atomic sphere is scaled down by a factor of 0.7 from the van der Waals radius.

**Fig. 3:**
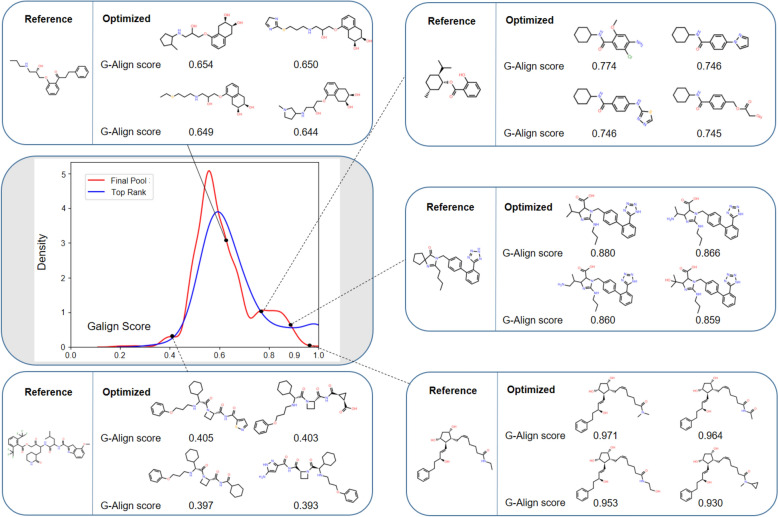
Distribution of molecules obtained by maximizing the atomic overlap score (G-Align) with each of the 200 reference molecules. The highest population appears near a score of 0.6, with molecules scoring below 1 still corresponding to chemically similar structures

The optimization results for G-Align, presented in Fig. 3, show that a range of chemicals with G-Align scores from 0.3 to 1.0 were obtained, with the highest population around 0.6, when 100 randomly selected molecules from each of DrugBank and BindingDB were used as reference molecules. Figure 3 also demonstrates that even a low G-Align score between 0.4 and 0.6 corresponds to chemically similar molecules. Results using the same CSA run parameters as CSearch are reported here, as varying the parameters did not result in qualitative changes. These findings suggest that, while BRICS-based virtual synthesis has limitations in exhaustively searching the chemical space, it serves as a reasonable baseline synthesis method for evaluating CSearch as a global chemical optimization protocol, as presented in this paper.

The objective function, the predicted docking score by the GNN, was also evaluated separately by examining the correlation between the surrogate GNN score and the GalaxyDock3 score. While the GNN model trained on database molecules showed high correlation with GalaxyDock3 scores, with *R*^2^ values of 0.872, 0.836, 0.826, and 0.863 for the four receptors MPro, BTK, ALK, and H1N1_NA, respectively, the optimized chemicals by CSearch showed reduced correlations of 0.273, 0.176, 0.234, and 0.476. This weak alignment between the surrogate model and the original scores suggests that a more suitable objective function is necessary for effective real-world chemical optimization [55]. Since developing improved measures of binding affinity or activity remains an active area of research, tests relying solely on docking methods may yield limited results. The effective global optimization performance of CSearch, as shown in the previous subsection, along with the analysis in this subsection, suggests that current limitations are more related to chemical scoring, which requires further research, than to chemical generation or optimization.

### Synthesizability of the molecules optimized by CSearch

**Fig. 4:**
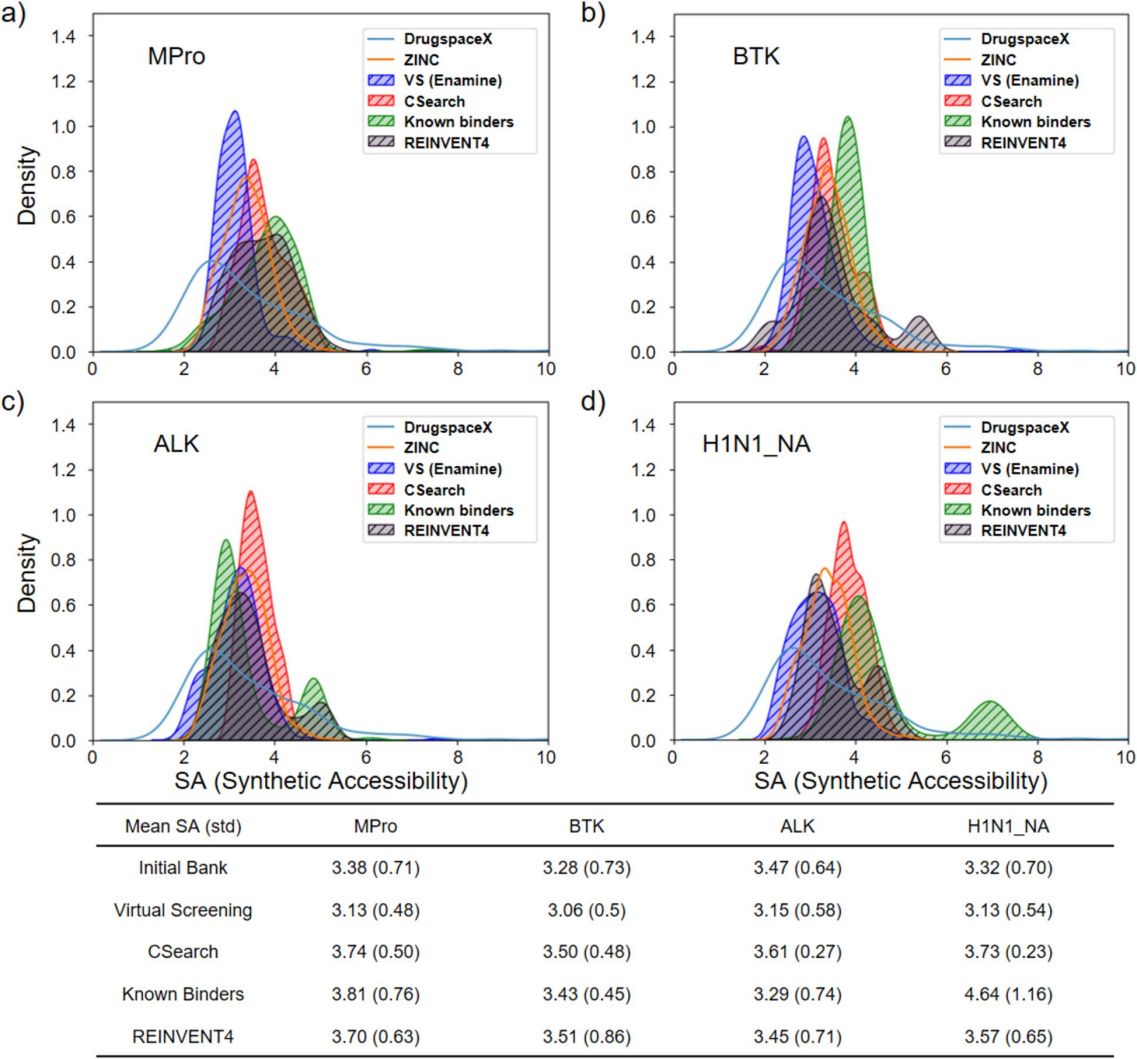
Distribution of SA scores of optimized chemicals and DB chemicals. Distribution of SA scores for the 300 chemicals optimized by CSearch (red) in comparison with the top 300 chemicals from virtual screening of Enamine HTS Collection (blue), top 300 chemicals from REINVENT4 (black), and 300 known binders (green) for four receptors, **a** Mpro, **b** BTK, **c** ALK, **d** H1N1_NA. In the background, SA distributions for DrugspaceX (skyblue) and ZINC chemicals (orange) are shown. In the table below, the mean SA and the standard deviation are presented

In general, there is no measure for chemical synthesizability as reliable as a human expert. Here, a common measure, the synthetic accessibility score [19] was employed. The synthesizability of the chemicals optimized by CSearch was examined in comparison with the top-scoring chemicals in the Enamine HTS Collection, those generated by REINVENT4, and known binders with high potency from BindingDB for the four receptors, as shown in Fig. 4. According to the figure, the SA scores for CSearch-optimized chemicals range from 2 to 5, similar to the known binders from binding DB. They are also within the SA distribution of DrugspaceX and ZINC. The CSearch-optimized chemicals show only slightly higher mean SA scores, by 0.4–0.7, than the same number of top-scoring library chemicals, which tend to have worse objective values (see Table 1).

### Diversity of the molecules optimized by CSearch

**Fig. 5:**
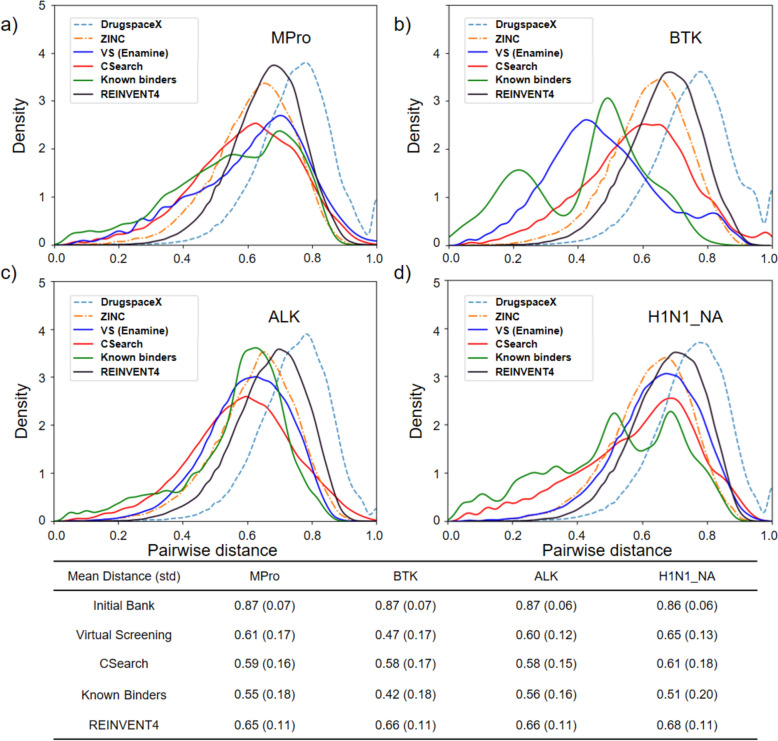
Distribution of pairwise distances of optimized chemicals and DB chemicals. Distribution of pairwise distances for the 300 chemicals optimized by CSearch (red) in comparison with the top 300 chemicals from virtual screening of the Enamine HTS Collection (blue), the top 300 generated chemicals from REINVENT4 (black), and 300 known binders (green) for four receptors **a** Mpro, **b** BTK, **c** ALK, **d** H1N1_NA. Distance distributions for DrugspaceX (dotted skyblue) and ZINC chemicals (dotted orange) are also shown. In the table below, the mean distance and standard deviation are presented

The internal diversity of the chemicals obtained by optimizing the given objective functions with CSearch was assessed by examining the pairwise Tanimoto distances of the chemicals from five independent runs of CSearch. According to Fig. 5, the distribution of the pairwise distances for the 300 CSearch-optimized chemicals is similar to that of the 300 known binders with high potency from BindingDB for all four receptors, ranging from 0.6 to 1. The top 300 chemicals from the Enamine HTS Collection show distance distributions similar to the optimized molecules in silico (CSearch) and the experimental binders (BindingDB) for MPro and BTK, for which the top library molecules show relatively better objective values (see Table 1).

Additionally, another diversity measure, termed ‘#Circles’ [56], was examined in Table 3. This metric represents the maximum number of exclusive spheres formed by molecules within a Tanimoto distance threshold of 0.7 in chemical space, reflecting chemical space coverage. The more optimized molecules generated by CSearch exhibit relatively low diversity compared to those obtained via virtual screening and REINVENT4, though the diversity is similar to that of known binders.

**Table 3:** Diversity measured by ‘#Circles’ for optimized compounds

Receptor	Virtual screening	REINVENT4	Known binders	CSearch
MPro	23	29	18	9
BTK	22	19	7	17
ALK	58	25	13	6
H1N1 NA	55	19	9	9

### Novelty of the molecules optimized by CSearch

**Fig. 6:**
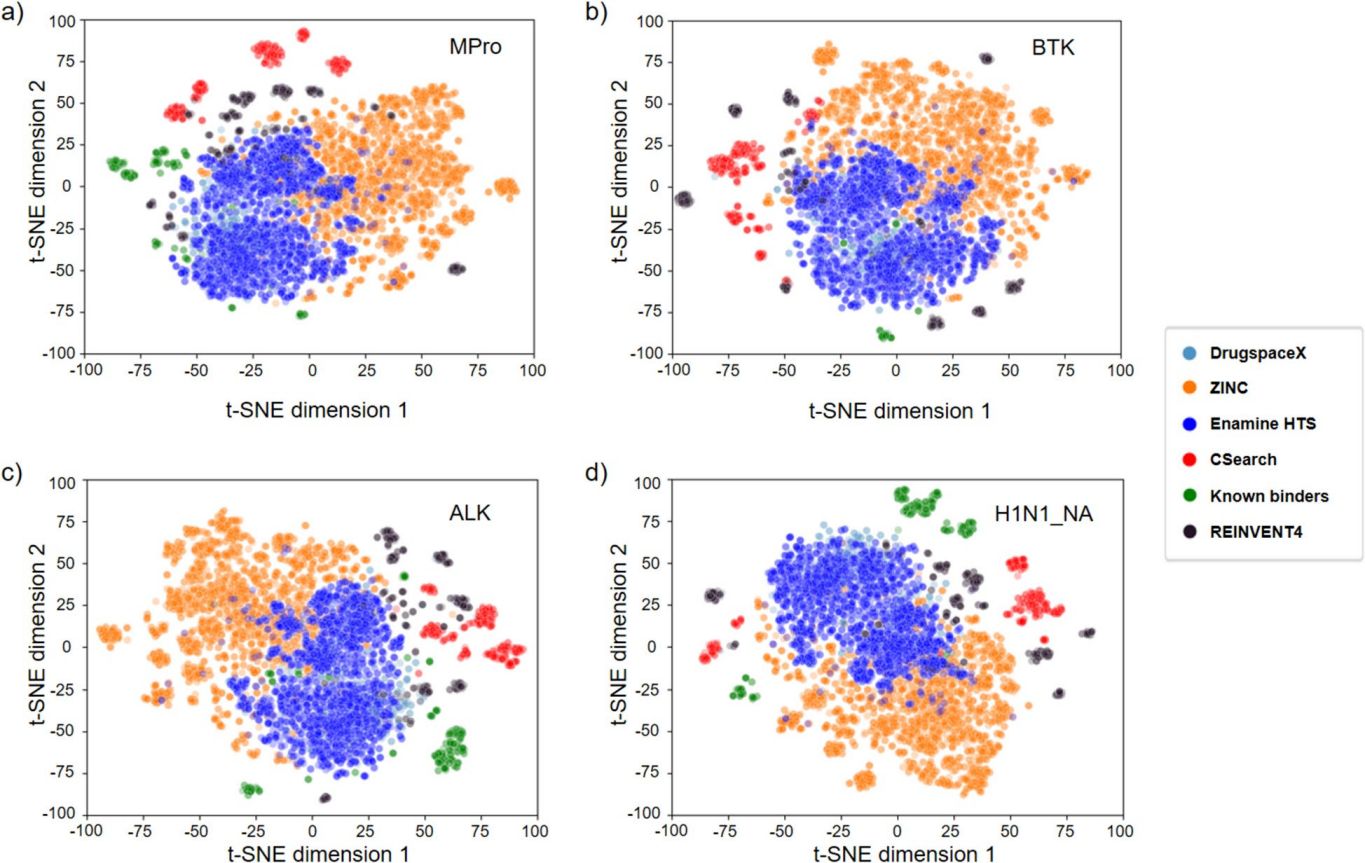
t-SNE plots representing relative distances of optimized chemicals and DB chemicals. Distributions of the 300 CSearch-optimized molecules (red), the top 300 molecules by REINVENT4 (black), and 300 known binders (green) for four receptors **a** Mpro, **b** BTK, **c** ALK, **d** H1N1_NA in the chemical space represented by two t-SNE dimensions. Distributions for DrugspaceX (skyblue), ZINC chemicals (orange) and Enamine HTS collection (deep colored blue) are also shown The optimized molecules and known binders appear in novel spots of the chemical space, except for the known binders for BTK

The novelty of the 300 chemicals obtained by CSearch optimization was compared with 300 known binders with high potency and the 300 chemicals generated by REINVENT4 in the chemical space represented by two t-SNE dimensions [57], as shown in Fig. 6. t-SNE plots made by TSNE module in scikit-learn. Chemicals generated by CSearch and REINVENT4, along with known binders belonged to different clusters from those in chemical databases, except for the known binders for BTK. This result illustrates that CSearch can explore novel areas in the chemical space that are not covered by existing databases in the process of extensive optimization of a given objective function.

### Examples of the optimized molecules

**Fig. 7:**
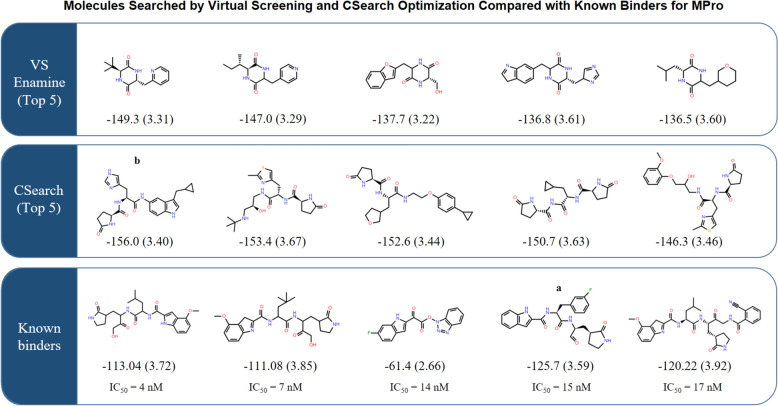
Two-dimensional structures of top 5 chemicals obtained by VS, CSearch, and Known binders for MPro. The molecular 2D structures of the top 5 chemicals obtained by virtual screening (VS) and CSearch for MPro (SARS-CoV-2 main protease) are compared with known binders with high potency. The objective value calculated in this study, with the synthetic accessibility score in parentheses, is shown below each molecule. IC_50_ values are also presented for the known binders

The top 5 molecules obtained by virtual screening (VS) of the Enamine HTS Collection and the top 5 by CSearch for the target MPro (SARS-CoV-2 main protease) were compared with five known binders with high potency, as shown in Fig. 7. Although a surrogate objective function was used, the CSearch-optimized molecules show similar overall size, shape, sub-structures, and functional groups to known binders. The virtual library screening resulted in smaller-sized molecules, which is consistent with their lower synthetic accessibility, as examined in Fig. 4. This implies a potential for CSearch to generate synthesizable, diverse, and novel compounds with highly optimized properties when combined with an objective function well-designed for a particular problem. The 2D chemical structures of the top molecules for the three other receptors are provided in Supporting Information (Additional file 1: Figure S2 to S4).

**Fig. 8:**
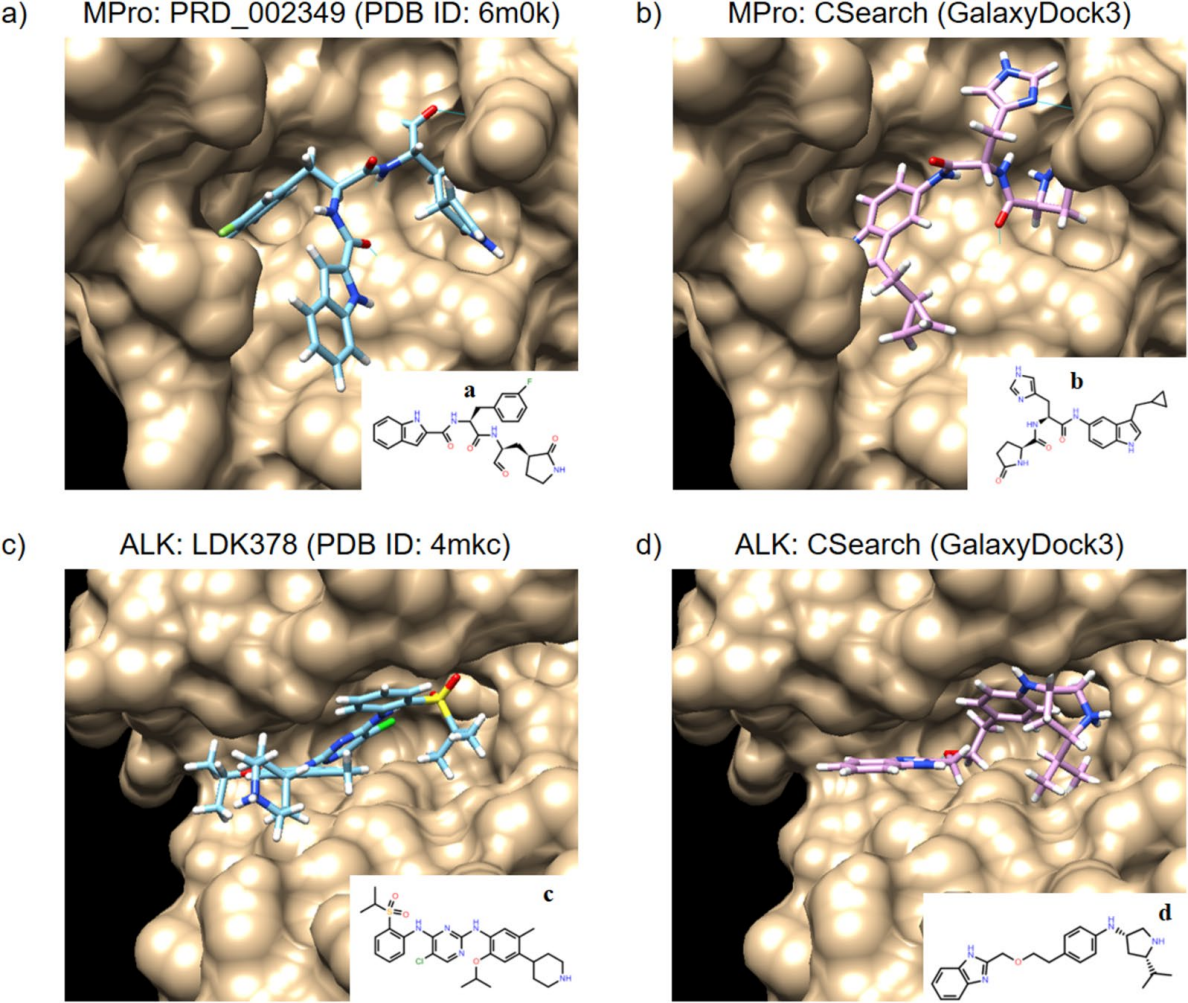
Binding poses of known binders and CSearch-optimized molecules for MPro and ALK. Binding poses of a known binder **‘a’** and a CSearch-optimized molecule **‘b’** for SARS-CoV-2 main protease (MPro) are shown in **a**, **b**, respectively, while the poses for a known binder **‘c’** and a CSearch-optimized molecule **‘d’** for anaplastic lymphoma kinase (ALK) are shown in **c**, **d**. The complex structures in **b**, **d** were generated by docking with GalaxyDock3 [43]

The molecules labeled as ‘a’ (a known binder) and ‘b’ (the top 1 molecule by CSearch) were compared in their complex structures with the receptor MPro in Fig. 8a, b. The binding pose of ‘b’ to the receptor structure, obtained by docking with GalaxyDock3 [44], is very similar to the experimental pose of the known binder. In Fig. 8c, d, the experimental complex structure of ‘c’ (a known binder) with the receptor ALK is compared with the predicted binding pose of ‘d’ (optimized by CSearch), also presenting very similar poses. It is intriguing that CSearch could generate molecules with reasonable predicted binding poses, even with a simplified objective function that does not directly account for the 3D binding poses.

## Conclusions

CSearch demonstrated significantly higher computational efficiency compared to virtual screening of chemical databases and a reinforcement learning-based method, REINVENT4 in optimizing the objective functions for four protein targets. The chemicals generated not only optimized the objective functions but also exhibited synthesizability and diversity comparable to those of chemical databases and known binders. Additionally, CSearch-optimized chemicals were highly novel and displayed binding poses to the receptors similar to known binders, underscoring CSA as an effective method for de novo molecule generation.

CSearch serves as an effective baseline model for rigorously evaluating the generation of molecules optimized for specific objective functions, guiding the development of methods that simultaneously evaluate and generate drug-like molecules. It is versatile enough to be applied to various drug-like property scores beyond the objectives presented here, ensuring synthetic accessibility and maintaining diversity among the generated chemicals. Consequently, as more accurate or desirable molecular property prediction models are developed, they can be integrated with CSearch to generate molecules with enhanced performance.

## Supplementary Information

Additional file 1: Method S1. CSA parameter optimization. Method S2. t-SNE plot parameters used in Figure 6. Method S3. GNN architecture for the objective function tested in CSearch. Table S1. Input atom node and bond edge features for GNN. Table S2. Hyperparameters and training configurations. Table S3. R2 of the trained GNN for the training, validation, and test sets. Table S4. Comparison of fragment selection in CSearch trial molecule generation with and without log frequency weighting. Figure S1. BRICS retrosynthesis rules used in virtual synthesis. Figure S2. Two-dimensional structures of top 5 chemicals obtained by VS, CSearch, and Known binders for BTK. Figure S3. Two-dimensional structures of top 5 chemicals obtained by VS, CSearch, and Known binders for ALK. Figure S4. Two-dimensional structures of top 5 chemicals obtained by VS, CSearch, and Known binders for H1N1_NA.

## Data Availability

The CSearch source code and datasets used in this article are available at https://github.com/seoklab/CSearch.git.

## Abbreviations

VS: Virtual screening
CSA: Chemical space annealing or conformational space annealing
MPro: SARS-CoV-2 main protease
BTK: Bruton tyrosine kinase
ALK: Anaplastic lymphoma kinase
H1N1_NA: H1N1 neuraminidase
SMILES: Simplified molecular-input line-entry system
