# Supplementary material for: CSearch: chemical space search via virtual synthesis and global optimization
Source: J Cheminform. 2024 Dec 5;16:137. doi: 10.1186/s13321-024-00936-8 (PMC11622599; doi:10.1186/s13321-024-00936-8)
Supplement: Supplementary file 1 — Additional file 1: Method S1. CSA parameter optimization. Method S2. t-SNE plot parameters used in Figure 6. Method S3. GNN architecture for the objective function tested in CSearch. Table S1. Input atom node and bond edge features for GNN. Table S2. Hyperparameters and training configurations. Table S3. R2 of the trained GNN for the training, validation, and test sets. Table S4. Comparison of fragment selection in CSearch trial molecule generation with and without log frequency weighting. Figure S1. BRICS retrosynthesis rules used in virtual synthesis. Figure S2. Two-dimensional structures of top 5 chemicals obtained by VS, CSearch, and Known binders for BTK. Figure S3. Two-dimensional structures of top 5 chemicals obtained by VS, CSearch, and Known binders for ALK. Figure S4. Two-dimensional structures of top 5 chemicals obtained by VS, CSearch, and Known binders for H1N1_NA. [file 13321_2024_936_MOESM1_ESM.docx]

**Supporting Information
CSearch: Chemical Space Search via Virtual Synthesis and Global Optimization**

Hakjean Kim^1^, Seongok Ryu^2^, Nuri Jung^1^, Jinsol Yang,^2^* and Chaok Seok^1, 2^*

*^1^Department of Chemistry, Seoul National University, Seoul 08826, Republic of Korea*

*^2^Galux Inc, Seoul 08738, Republic of Korea*

**Method S1. CSA parameter optimization**

**Method S2. t-SNE plot parameters used in Figure 6**

**Method S3. GNN architecture for the objective function tested in CSearch**

Table S1. Input atom node and bond edge features for GNN

Table S2. Hyperparameters and training configurations

**Table S3. *R*^2^ of the trained GNN for the training, validation, and test sets**

**Table S4. Comparison of fragment selection in CSearch trial molecule generation with and without log frequency weighting**

**Figure S1. BRICs retrosynthesis rule used in virtual synthesis**

**Figure S2. Two-dimensional structures of top 5 chemicals obtained by VS, CSearch, and Known binders for BTK.**

**Figure S3. Two-dimensional structures of top 5 chemicals obtained by VS, CSearch, and Known binders for ALK.**

**Figure S4. Two-dimensional structures of top 5 chemicals obtained by VS, CSearch, and Known binders for H1N1_NA.**

**Method S1. CSA parameter optimization**

A rather large bank size of n = 60 was determined to be necessary to generate diverse chemicals after testing sizes of 20, 40, 50, 60, 80, and 100. A small number of seeds (10% of bank) and seed cycles (2) were determined to be sufficient for effective objective optimization after testing 10%, 20%, 30%, 40%, and 50% of the bank, and 1, 2, 3, 4, and 5 seed cycles, respectively. The annealing schedule for *R*_cut_ followed the typical conformational space annealing approach, starting from half of the average distance in the initial bank and reaching a minimum value of four-tenths of the initial *R*_cut_ after *k* cycles. The values *k* = 20 and a maximum number of cycles = 50 resulted in proper synthesizability and diversity.

**Method S2. t-SNE plot parameters used in Figure 6**

The t-SNE plots in **Figure 5** were generated using the TSNE module in scikit-learn with the following parameters: n_components=2, perplexity=30.0, early_exaggeration=4.0, learning_rate=1000.0, n_iter=1000, metric='euclidean', and init='random' (default TSNE settings in scikit-learn). Morgan fingerprints were obtained using AllChem.GetMorganFingerprintAsBitVect in the RDKit module with radius=2 and nbits=2048. All other parameters were set to RDKit’s default values.

**Method S3. GNN architecture for the objective function tested in CSearch**

**Table S1. Input atom node and bond edge features for GNN**

| Node features | | | |
| --- | --- | --- | --- |
| Type | **Values** | **Dimension** | **Representation** |
| Atom type | 'C', 'N', 'O', 'S', 'F', ‘H', 'Si', 'P', 'Cl', 'Br', ’Li', 'Na', 'K', 'Mg', 'Ca', 'Fe', 'As', 'Al', 'I', 'B','V', 'Tl', 'Sb', 'Sn', 'Ag', 'Pd', 'Co', 'Se', 'Ti', 'Zn', 'Ge', 'Cu', 'Au', 'Ni', 'Cd', 'Mn', 'Cr', 'Pt', 'Hg', 'Pb' | 40 | One-hot |
| Aromatic | 0, 1 | 1 | True or false |
| Implicit valence | 0, 1, 2, 3, 4, 5 | 6 | One-hot |
| All hydrogen atom | 0, 1, 2, 3, 4 | 5 | One-hot |
| Degree | 0, 1, 2, 3, 4, 5 | 6 | One-hot |
| Edge features | | | |
| Type | **Values** | **Dimension** | **Representation** |
| Bond type | Single, Double, Triple, Aromatic | 4 | One-hot |
| Ring | 0, 1 | 1 | True or false |
| Conjugated | 0, 1 | 1 | True or false |

We trained graph neural networks (GNNs) to reproduce GalaxyDock3 energy of ligands binding to the four receptors. By using SMILES representation and RDKit built-in functions as described in **Table S1**, we assigned a set of initial node descriptors $X=\{x_{i}\}$ and a set of edge descriptors $E=\{e_{ij}\}$ to generate the graph representation $G(X,E)$ of given molecule, where $x_{i}\in\mathcal{R}^{d_{n}}$ denotes the *i*-th node descriptor and $e_{ij}\in\mathcal{R}^{d_{e}}$ denotes the edge descriptor between *i*-th and *j*-th node if two nodes were connected by chemical bond. Then, the first embedding layer of GNNs linearly transformed the initial vectors to the pre-defined node and edge feature dimension $d$:

$$h_{i}^{(0)}=W_{n,emb}x_{i}$$

$$e_{ij}^{(0)}=W_{e,emb}e_{ij},$$

where $W_{n,emb}\in\mathcal{R}^{d \times d_{n}}$ and $W_{n,emb}\in\mathcal{R}^{d \times d_{e}}$ are weight parameters, and $d_{n}$, $d_{e}$, and $d$ are node, edge and transformed feature dimensions.

Then, the *l*-th node update layers, for $l\in\left\{ 1, \ldots, L \right\}$, update the node features from $h^{(l-1)}\in\mathcal{R}^{(l-1)}$ to $h^{(l-1)}\in\mathcal{R}^{(l)}$ as follows:

$$h_{i}^{(l)}=\mathrm{DO}\left( \mathrm{LN}\left( h_{i}^{(l-1)}+MLP\left( \sum_{j\in\mathcal{N}_{i}} h_{j}^{(l-1)}+e_{ij}^{(l-1)} \right) \right) \right),$$

where DO and LN stands for dropout [1] and layer normalization [2], and MLP is two-layer multi-layer perceptron with non-linear activation $\sigma$:

$$\mathrm{ML}P^{\left( l \right)}\left( x \right)= W_{2}^{\left( l \right)}\sigma\left( W_{1}^{(l)}x+b_{1}^{(l)} \right)+b_{2}^{(l)} .$$

After updating the node features with a stack of *L* node update layers, we aggregated the updated node features $h_{i}^{(L)}$ by using pooling by multi-head attention (PMA) [3], which assigned different atom-wise importance to each node:

$$z_{G}= \sum_{v} \alpha_{v}h_{v}^{(L)},$$

where $\alpha_{v}$ is the atom-wise importance assigned to the *v*-th node given by

$$\alpha_{v}=\mathrm{softmax}\left( \frac{1}{\sqrt{d}}\mathbf{1}\left( W_{pma}h_{v}^{(L)} \right)^{T} \right),$$

where $W_{pma}\in\mathcal{R}^{d\times d}$ is a weight parameter and $1\in\mathcal{R}^{d}$ is a one-initialized seed vector of size *d*.

Finally, for the *n*-th sample in the training dataset predictive energy score  $\hat{y}_{n}$ and its uncertainty $\hat{\sigma}_{n}$ were obtained by the prediction layer:

$$\hat{y}_{n}=W_{m}^{T}z_{G_{n}}+b_{m}$$

$$\hat{\sigma}_{n}=W_{u}^{T}z_{G_{n}}+b_{u},$$

where $W_{m}^{T}$, $W_{u}^{T}$, $b_{m}$, and $b_{u}$ are weight and bias parameters.

For gradient-descent minimization of loss function, we used heteroscedastic mean-squared error loss,

$$\mathcal{L}\left( \left\{ y_{n}, \hat{y}_{n}, \hat{\sigma}_{n} \right\} \right)=\frac{1}{N}\sum_{n=1}^{N} \frac{1}{2\hat{\sigma}_{n}^{2}}\left( y_{n}- \hat{y}_{n} \right)^{2}+\frac{1}{2}\log\hat{\sigma}_{n}^{2}$$

where $y_{n}$ is the ground-truth energy score and $N$ is the size of training dataset, to estimate both predictive energy score $\hat{y}_{n}$ and its uncertainty $\hat{\sigma}_{n}$.

We used PyTorch [4] and DeepGraphLibrary [5] for implementation of GNN models. The hyperparameters and training configurations are detailed in **Table S2**.

**Table S2. Hyperparameters and training configurations**

| Number of node update layers (*L*) | 4 |
| --- | --- |
| Node/edge feature dimension (d) | 128 |
| Number of attention heads in PMA | 4 |
| Number of training epochs | 150 |
| Optimizer | AdamW |
| Batch size | 64 |
| Learning rate at 0/40/80/120 epoches | 10^-3^/10^-4^/10^-5^/10^-6^ |
| Dropout probability | 0.2 |
| Weight decay | 10^-6^ |

**Table S3. *R*^2^ of the trained GNN for the training, validation, and test sets**

| **Receptors / Sets** | Training | Validation | Test |
| --- | --- | --- | --- |
| MPro | 0.852 | 0.686 | 0.872 |
| BTK | 0.818 | 0.845 | 0.836 |
| ALK | 0.814 | 0.837 | 0.826 |
| H1N1 NA | 0.839 | 0.838 | 0.863 |

**Table S4. Comparison of fragment selection in CSearch trial molecule generation with and without log frequency weighting**

| CSearch | Applying Weighting | | | No weighting | | |
| --- | --- | --- | --- | --- | --- | --- |
|  | Top 1 obj | Mean obj | Mean SA | Top 1 obj | Mean obj | Mean SA |
| MPro | -156.5 | -141.14 | **3.87** | **-157.1** | **-144.6** | 4.46 |
| BTK | -211.5 | **-181.99** | **3.82** | -224.1 | -190.9 | 3.91 |
| ALK | **-173** | **-153.47** | **3.95** | -159.5 | -139.75 | 4.23 |
| H1N1_NA | **-153.1** | **-142.96** | **4.108** | -146.4 | -137.67 | 4.29 |


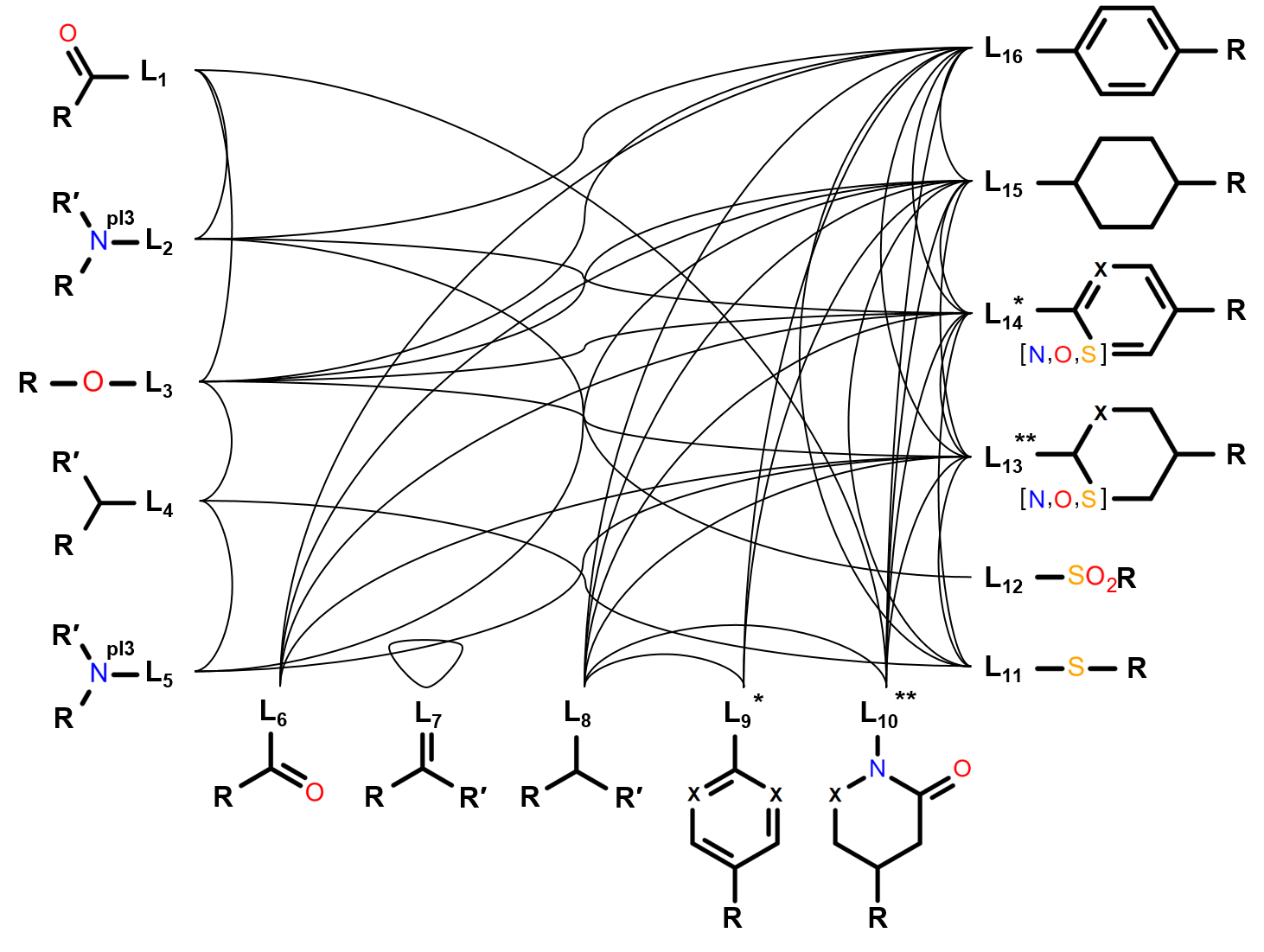


**Figure S1. BRICS retrosynthesis rules used in virtual synthesis.** To trace the origins of medicinal chemistry, carbonyl and alkyl fragments (L_1_ / L_6_, L_4_ / L_8_) are represented as independent group but identical. The R groups may include additional linking atoms or serve as linking atoms themselves, while R' groups may consist of hydrogen only. 'X' denotes any element from the set C, N, O, or S.

*: Although the term generally refers to all aromatic rings, it is represented here as benzene-based for simplicity.

**: Although the term generally refers to all single bond rings, it is represented here as hexane-based for simplicity.


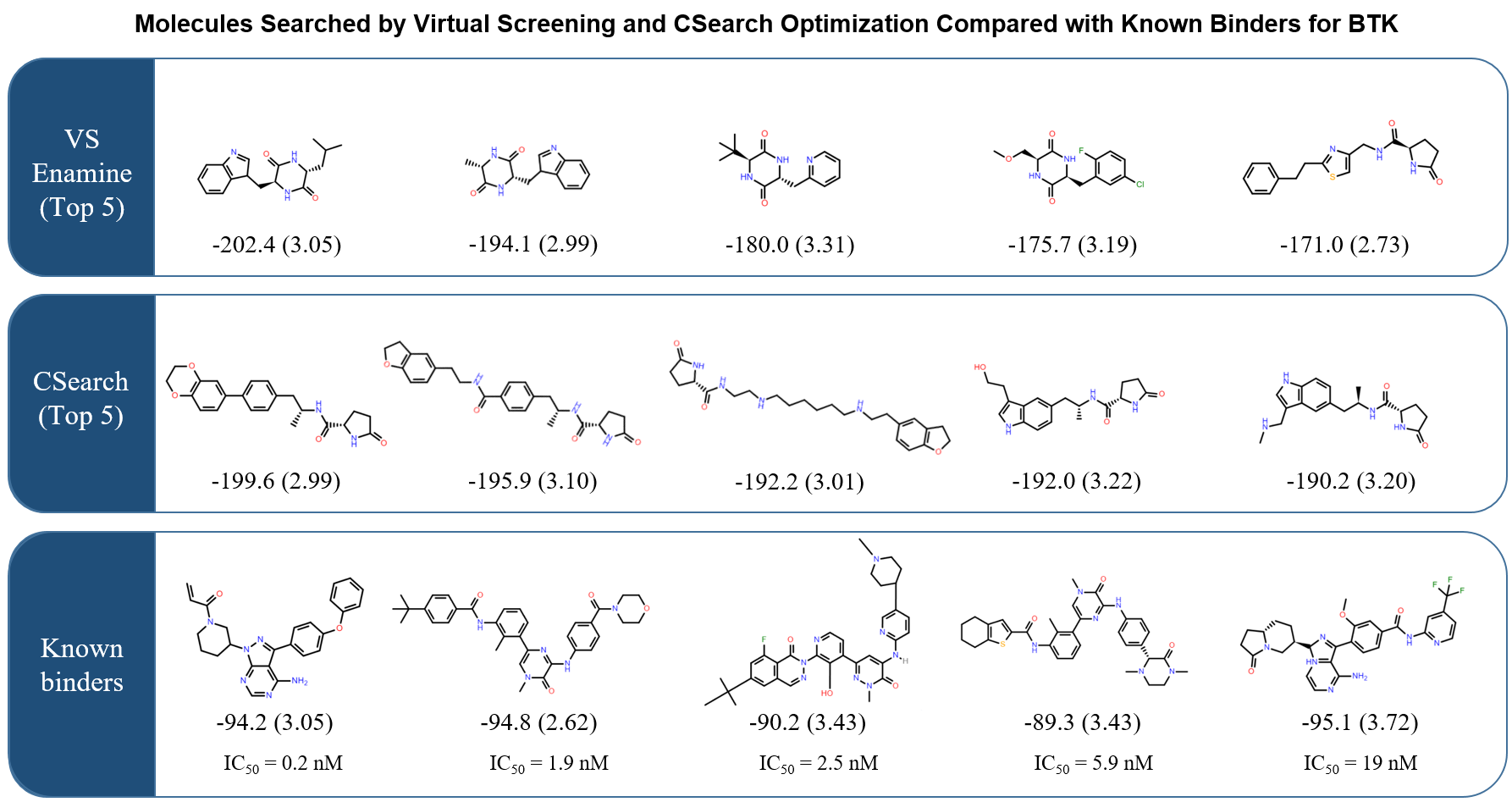


**Figure S2. Two-dimensional structures of top 5 chemicals obtained by VS, CSearch, and Known binders for BTK.**


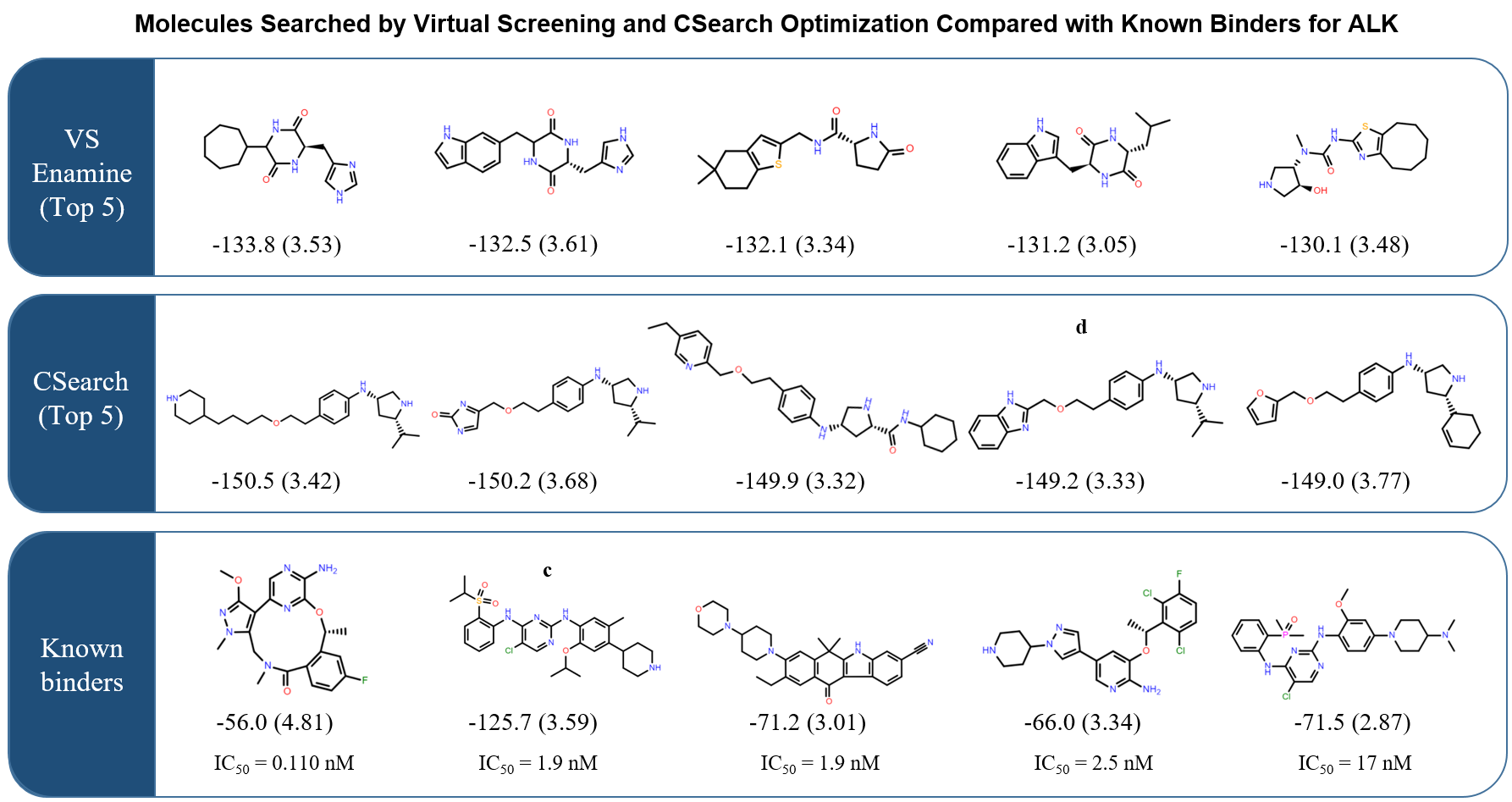


**Figure S3. Two-dimensional structures of top 5 chemicals obtained by VS, CSearch, and Known binders for ALK.**


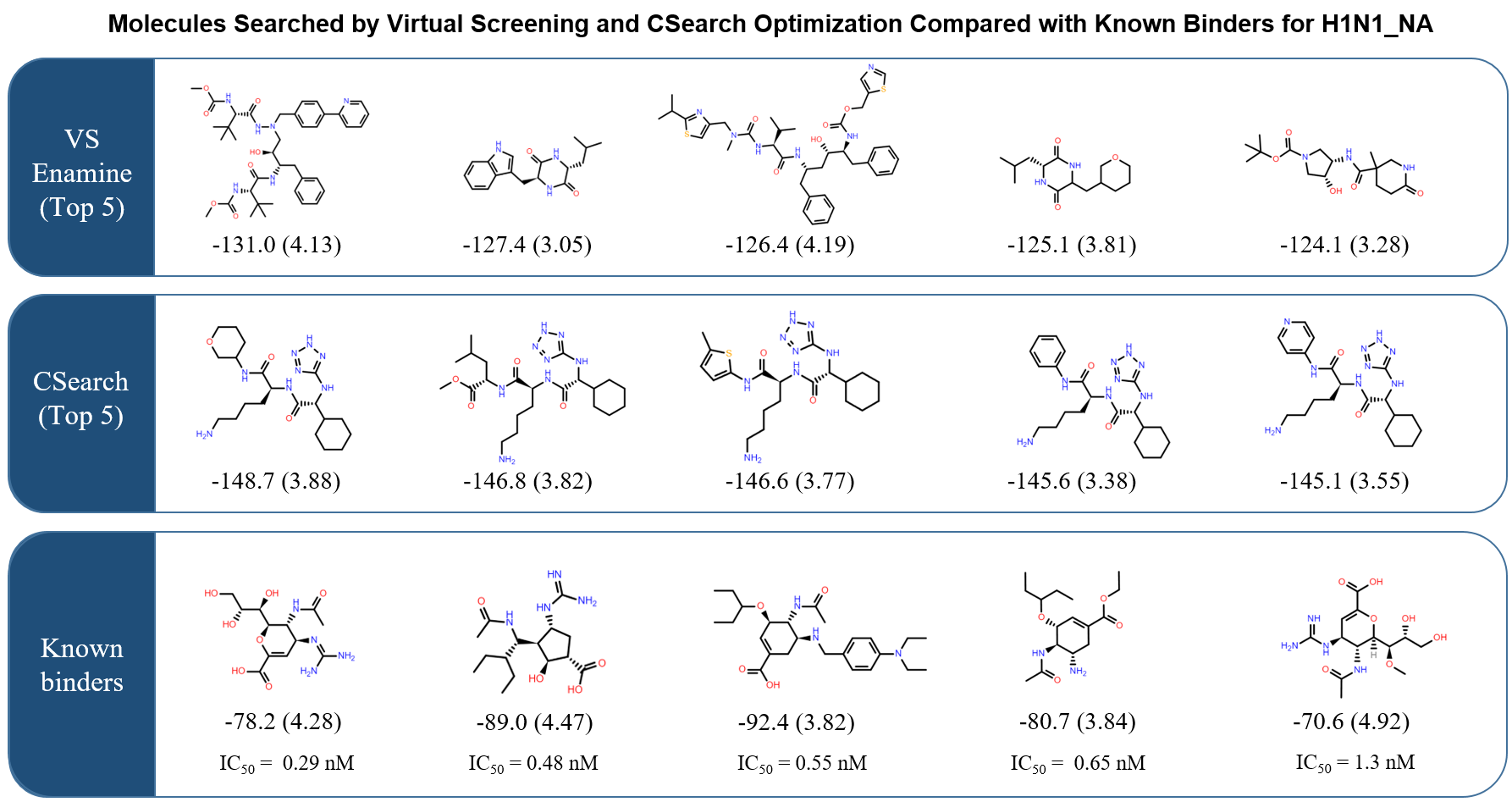


**Figure S4. Two-dimensional structures of top 5 chemicals obtained by VS, CSearch, and Known binders for H1N1_NA.**

**References**

1. Ba JL, Kiros JR, Hinton GE (2016) Layer normalization. arXiv preprint arXiv:1607.06450

2. Lee J, Lee Y, Kim J et al. (2019) Set transformer: A framework for attention-based

permutation-invariant neural networks. In: International conference on machine learning.

PMLR, p 3744-3753

3. Paszke A, Gross S, Chintala S et al. (2017) Automatic differentiation in pytorch.

4. Srivastava N, Hinton G, Krizhevsky A et al. (2014) Dropout: a simple way to prevent neural

networks from overfitting. The journal of machine learning research 15:1929-1958

5. Wang MY (2019) Deep graph library: Towards efficient and scalable deep learning on

graphs. In: ICLR workshop on representation learning on graphs and manifolds.
